# Use of pharmacotherapy for alcohol use disorder in Manitoba, Canada: A whole-population cohort study

**DOI:** 10.1371/journal.pone.0257025

**Published:** 2021-09-03

**Authors:** Geoffrey Konrad, Christine Leong, James M. Bolton, Heather J. Prior, Michael T. Paillé, Josh Nepon, Deepa Singal, Okechukwu Ekuma, Jennifer E. Enns, Nathan C. Nickel

**Affiliations:** 1 Department of Psychiatry, Rady Faculty of Health Sciences, University of Manitoba, Winnipeg, Canada; 2 College of Pharmacy, Rady Faculty of Health Sciences, University of Manitoba, Winnipeg, Canada; 3 Manitoba Centre for Health Policy, Department of Community Health Sciences, Rady Faculty of Health Sciences, University of Manitoba, Winnipeg, Canada; 4 Dept. of Community Health Sciences, Rady Faculty of Health Sciences, University of Manitoba, Winnipeg, Canada; McGill University, CANADA

## Abstract

**Objective:**

Update the evidence on use of pharmacotherapy for alcohol use disorder in a Canadian population.

**Methods:**

Using whole-population administrative data from Manitoba, Canada, we identified all residents age 12+ who were first diagnosed with alcohol use disorder between April 1, 1996 and March 31, 2015, and compared characteristics of those who filled a prescription for naltrexone, acamprosate or disulfiram at least once during that period to those who did not fill a prescription for an alcohol use disorder medication.

**Results:**

Only 1.3% of individuals with alcohol use disorder received pharmacotherapy (62.3% of prescriptions were for naltrexone, 39.4% for acamprosate, 7.5% for disulfiram). Most prescriptions came from family physicians in urban alcohol use disorder (53.6%) and psychiatrists (22.3%). Individuals were more likely to fill a prescription for alcohol use disorder medication if they lived in an urban vs rural environment (OR 2.25; 95% CI 1.83–2.77) or had a mood/anxiety disorder diagnosis vs no diagnosis (OR 2.40, 95% CI 1.98–2.90) in the five years before being diagnosed with alcohol use disorder.

**Conclusion:**

Despite established evidence for the effectiveness of pharmacotherapy for alcohol use disorder, these medications continue to be profoundly underutilized in Canada.

## Introduction

Alcohol use disorder (AUD) is characterized by compulsive alcohol use and loss of control over alcohol intake, and it is associated with substantial morbidity and mortality in North American populations [1–3]. In Canada, more than 3000 deaths/year are attributable to alcohol consumption [3]. Globally, 5.3% of all deaths and 5.0% of the global burden of disease and injury (measured in Disability-Adjusted Life Years) in 2016 have been attributed to alcohol, translating into approximately 3 million alcohol-attributable deaths after taking into account the potential benefits of low-risk drinking [4]. Alcohol use is directly related to many other adverse physical and psychosocial health outcomes, including cancer, infectious disease, cardiovascular disease, neuropsychiatric disease, and intentional and unintentional self-injury [4–6]. Moreover, alcohol drinking affects not only individuals, but has a negative impact on society as a whole, as it is often associated with increased rates of child abuse and/or neglect [7–9], intimate partner violence [10, 11], and hospitalization and mortality rates [12–14].

Despite the substantial individual, societal, and global public health burden of AUD, it appears to be largely untreated [1, 15]. Even though effective pharmacotherapies for AUD have been available for decades and are supported by numerous systematic reviews and meta-analyses establishing both their efficacy and safety [16–19], drugs such as naltrexone and acamprosate have been found to be severely underutilized among AUD patients in select populations [20–22]. For example, in studies of US military veterans with AUD, fewer than 5% received any kind of pharmacotherapy [21, 22]. In another study enrolling low-income AUD patients in Ontario, Canada, the rate of AUD medications dispensed was similarly low [23]. While these low rates are concerning, questions as to the generalizability of previous findings remain, since many of the existing studies examining AUD pharmacotherapy enrolled non-representative samples. Thus, the rate of treatment uptake in the general population is still largely unknown.

Our study aims to address this gap in the literature, leveraging comprehensive whole-population data to determine the proportion of individuals receiving pharmacotherapy for AUD. We used administrative data from the general population of Manitoba, Canada to examine the use of pharmacotherapy (naltrexone, acamprosate, and/or disulfiram) among individuals with AUD. We describe their sociodemographic and clinical characteristics and their healthcare use, determine the medical specialties most likely to prescribe them AUD medications, and identify variables that predict whether or not individuals with AUD are likely to fill prescriptions for AUD pharmacotherapy. The study’s findings shed light on prescribing patterns among physicians caring for AUD patients, and inform clinical practice for treating AUD in general populations.

## Methods

### Study setting

Manitoba is a central Canadian province with approximately 1.3 million residents, and the main urban centre is the city of Winnipeg (population approx. 780,000). The province is representative of Canada in many respects, ranking in the middle for most indicators of health and education [24, 25]. Manitoba also has the capacity to link individual-level information on prescription medications to large population-based administrative datasets, which contain information on virtually all contacts with the healthcare system and social services over the last 20+ years. All residents of Manitoba have universal healthcare coverage.

### Data sources

This study used data from the population-based Manitoba Population Research Data Repository housed at the Manitoba Centre for Health Policy at the University of Manitoba, Canada. The Repository contains 70+ databases with information from the health, education, social, and justice sectors on nearly all residents of the province of Manitoba (except for active military personnel and incarcerated persons), in many cases going back more than 40 years. The data are de-identified (names and addresses removed), but can be linked across databases and over time using a unique numeric personal identification code.

The databases we used in this study include the Manitoba Health Insurance Registry (age, sex, region of residence, mortality), the Drug Program Information Network (DPIN) database (prescription medications, prescriber information), the Hospital Abstracts Database (hospitalizations, diagnosis codes), the Medical Services Database (ambulatory visits, diagnosis codes), Canada Census data (income quintile), and Vital Statistics (cause of death). Validity checks have demonstrated that these datasets have first-rate linkage accuracy [26, 27].

### Study population

Using the administrative data, we identified all people in the province of Manitoba over the age of 12 who received a first diagnosis of AUD within the study period (April 1, 1996 –March 31, 2015). For the specific definition of AUD we used, see **S1 Table**. The date an individual first met the criteria for an AUD diagnosis was considered their ‘index date’. Within this cohort, we compared individuals diagnosed with AUD who received at least one prescription for naltrexone (ATC N07BB04), acamprosate (ATC N07BB03), or disulfiram (ATC N07BB01) and who had at least five years of continuous health coverage prior to their index date, to individuals diagnosed with AUD who did not fill any prescriptions during the study period.

### Outcome variables

The study outcomes included: the proportion of individuals with AUD who filled a prescription for AUD pharmacotherapy, sociodemographic and clinical characteristics of individuals with AUD and the medical specialties who most often prescribed AUD medications. We used records of prescriptions from the DPIN database, including drug identification number, date of dispensation, and prescriber specialty, to identify individuals with AUD who were treated with pharmacotherapy. Data on the sociodemographic characteristics of the study population came from Repository datasets listed above. Resource Utilization Bands (RUBs) are a simplified ranking system of overall morbidity across six categories; individuals who would be expected to use the same level of resources are grouped together [28]. Individuals diagnosed with a substance use disorder, personality disorder, or psychosis in the five years before index date were identified using ICD codes (see **S2 Table**), and those diagnosed with mood and anxiety disorders in the five years before index date were identified using a combination of ICD codes and ATC codes (**S3 Table**). The clinical and healthcare use characteristics we examined included number of hospitalizations, ambulatory visits and emergency department visits in the year before AUD diagnosis, and we also looked at specifically at how many of the hospitalizations and ambulatory visits were related to mental health. We examined the number of prescriptions individuals with AUD filled at least twice consecutively and the specific types of medications they received in the year before being diagnosed (see **S4 Table** for ATC codes). Lastly, we ran a multivariate logistic regression model to determine whether age, sex, urban/rural geography and mental health were important predictors of receiving AUD pharmacotherapy. These variables were selected based on evidence of their contribution driving difference in medication use and access to care described in the literature [29–31].

### Statistical analysis

Two-sided Chi-squared tests and t-tests for unequal variances were used to identify statistically significant (p<0.05) differences in categorical and continuous outcomes, respectively, between the two groups. We calculated the proportion of each medical specialty who prescribed AUD medications. We ran multivariable hierarchical regression models to identify significant predictors of receiving pharmacotherapy for AUD; individual-level factors included age, sex (female reference), urban residence, and co-occurring mood or anxiety disorders. Individuals were clustered by neighbourhood (urban-dwelling individuals) and regional health district (rural-dwelling individuals).

### Ethics

This study was approved by the University of Manitoba Human Research Ethics Board and Health Information Privacy Committee at Manitoba Government Department of Health.

## Results

The proportion of Manitobans with AUD using pharmacotherapy is presented in **Table 1.** Between 1996 and 2015, 37,388 individuals were diagnosed with AUD, representing 2.9% of the Manitoba population. Among these, 493 individuals received a prescription medication for AUD and 36,895 individuals did not receive any AUD-specific pharmacotherapy. Among individuals who received pharmacotherapy for AUD, 58.0% were prescribed naltrexone, 36.3% were prescribed acamprosate, and 5.7% were prescribed disulfiram. A higher proportion of males vs females filled a prescription for each type of AUD medication. **Table 1** also presents the sociodemographic characteristics of the study cohort. The mean age at index date for both groups was 40 years. More than half of individuals who filled a prescription for an AUD medication were first diagnosed between the ages of 30–50, while non-prescription users were more evenly distributed among age groups from 12–79 years. The majority of those who filled a prescription lived in urban vs rural settings, and they were fairly evenly represented across income quintiles, whereas those who did not fill a prescription demonstrated a clear income gradient.

**Table 1:** Sociodemographic characteristics of Manitobans with alcohol use disorder.

	Filled Prescription for AUD Medication	Did Not Fill Prescription for AUD Medication	p-value*
**USE OF PHARMACOTHERAPY**			
** N (% of total)**	**493 (1.3)**	**36,895 (98.6)**	
** Filled prescription for naltrexone, n (%)**	**286 (58.0)**	--	--
** Male**	154 (53.8)		
** Female**	132 (46.2)		
** Filled prescription for acamprosate, n (%)**	**179 (36.3)**	--	--
Male	108 (60.3)		
** Female**	71 (39.7)		
** Filled prescription for disulfiram, n (%)**	**28 (5.7)**	--	--
Male	22 (78.6)		
** Female**	6 (21.4)		
**SOCIODEMOGRAPHIC CHARACTERISTICS**			
**Mean Age (years)**	40.02	40.48	0.419
**Age Group, n (%)**			**<0.0001**
12–19	16 (3.3)	5,369 (14.6)	
20–29	91 (18.5)	8,223 (22.3)	
30–39	137 (27.8)	6,213 (16.8)	
40–49	136 (27.6)	5,588 (15.2)	
50–59	88 (17.9)	4,547 (12.3)	
60–69	16 (3.3)	3,281 (8.9)	
70+	9 (1.8)	3,674 (9.9)	
**Sex (overall), n (%)**			**0.005**
Male	284 (57.6)	23,511 (63.7)	
Female	209 (42.4)	13,384 (36.3)	
**Residence, n (%)**			**<0.0001**
Urban	369 (74.9)	20,069 (54.4)	
Rural	124 (25.2)	16,826 (45.6)	
**Income Quintile, n (%)**			**<0.0001**
U1 (lowest)	67 (13.6)	7,399 (20.1)	
U2	81 (16.4)	4,186 (11.4)	
U3	79 (16.0)	3,330 (9.0)	
U4	68 (13.8)	2,599 (7.0)	
U5 (highest)	72 (14.6)	2,054 (5.6)	
R1 (lowest)	s	5,784 (15.7)	
R2	19 (3.9)	3,510 (9.5)	
R3	30 (6.1)	2,640 (7.2)	
R4	30 (6.1)	2,660 (7.2)	
R5 (highest)	32 (6.5)	2,148 (5.8)	
Income Not Available	s	585 (1.6)	

All index dates 1996/97 to 2014/15 unless otherwise noted. s: This result has been suppressed due to small numbers in this cell (1–5 individuals)–however, these individuals are still included in the overall analyses.

*p-value is for chi-squared tests (categorical outcomes) or t-tests (continuous outcomes).

**Table 2** presents the clinical characteristics and healthcare use of the study cohort. The majority of AUD medication prescription users fell into Band 3 or 4 on the RUB scale (moderate health resource use), while many non-prescription users were in Band 0 (no significant health resource use). Prescription users were more likely than non-prescription users to have a mood- or anxiety-related diagnosis leading up to their index date. Those who filled prescriptions for AUD medication had more hospitalizations and ambulatory visits in the year prior to index date, and were also more likely to have mental health-related ambulatory care visits than those who did not fill a prescription. Neither the number of emergency department visits nor the number of prescription medications for chronic conditions in the year prior to index date was different between the two groups. Prescription users were more likely than non-users to receive SSRI antidepressants, but not TCA antidepressants. They were also more likely to receive sedatives, anxiolytics and the anti-epileptic medication lamotrigine.

**Table 2:** Clinical characteristics and healthcare use of Manitobans with alcohol use disorder.

	Filled Prescription for AUD Medication	Did Not Fill Prescription for AUD Medication	p-value **
**CLINICAL CHARACTERISTICS**			
**Resource Utilization Band (morbidity), n (%)**			**<0.0001**
0 (non-user)	57 (11.6)	13,317 (36.1)	
1 (lowest)	14 (2.8)	1,578 (4.3)	
2	55 (11.2)	5,642 (15.3)	
3	295 (59.8)	13,441 (36.4)	
4	63 (12.8)	2,438 (6.6)	
5 (highest)	9 (1.8)	479 (1.3)	
**Comorbidities, n (%)**			
Substance Abuse Disorder (excl. alcohol)	54 (11.0)	3,243 (8.8)	0.0924
Mood and Anxiety Disorders	320 (64.9)	15,008 (40.7)	**<0.0001**
Personality Disorders	31 (6.3)	1,748 (4.7)	0.1082
Psychoses	23 (4.7)	2,359 (6.4)	0.1185
**HEALTHCARE USE**			
**# Hospitalizations in the year before index date, mean (SD)**	83 (0.86)	10,705 (0.81)	**<0.0001**
For mental health reasons	29 (0.37)	1,796 (0.32)	0.3119
**# Ambulatory Visits in the year before index date, mean (SD)**	4,239 (10.07)	240,361 (7.79)	**<0.0001**
For mental health reasons	1,630 (6.37)	48,679 (3.78)	**<0.0001**
**# Emergency Department Visits in the year before index date, mean (SD)** *	343 (2.18)	12,488 (2.27)	0.8103
**# Chronic Prescription Medications in the year before index date, mean (SD)**	819 (2.06)	57,623 (2.65)	0.0794
**Chronic Prescription Medication Groups, n (%)**			**<0.0001**
0	187 (37.9)	20,197 (54.7)	
1–3	232 (47.1)	10,771 (29.2)	
4–8	68 (13.8)	4,745 (12.9)	
9+	6 (1.2)	1,182 (3.2)	
**Use of Medications 1 year before index date (2+ Rx with gap < 25% days’ supply), n (%)**			
SSRI Antidepressants	121 (24.5)	3,238 (8.8)	**<0.0001**
TCA Antidepressants	14 (2.8)	1,065 (2.9)	0.9515
Other Antidepressants	80 (16.2)	2,038 (5.5)	**<0.0001**
Sedatives and Anxiolytics	118 (23.9)	5,228 (14.2)	**<0.0001**
Antipsychotics (excl. lithium)	31 (6.3)	1,656 (4.5)	0.0629
Anticonvulsants	17 (3.5)	845 (2.3)	0.0888
Lithium	s	180 (0.5)	0.3815
Opioids	30 (6.1)	2,911 (7.9)	0.1570

All index dates 1996/97 to 2014/15 unless otherwise noted. s: This result has been suppressed due to small numbers (1–5 individuals)–however, these individuals are still included in the analyses; SD: Standard deviation.

*Limited to Winnipeg residents only, index dates 2000/01-2012/13.

**p-value is for chi-squared tests (categorical outcomes) or t-tests (continuous outcomes).

**Fig 1:**
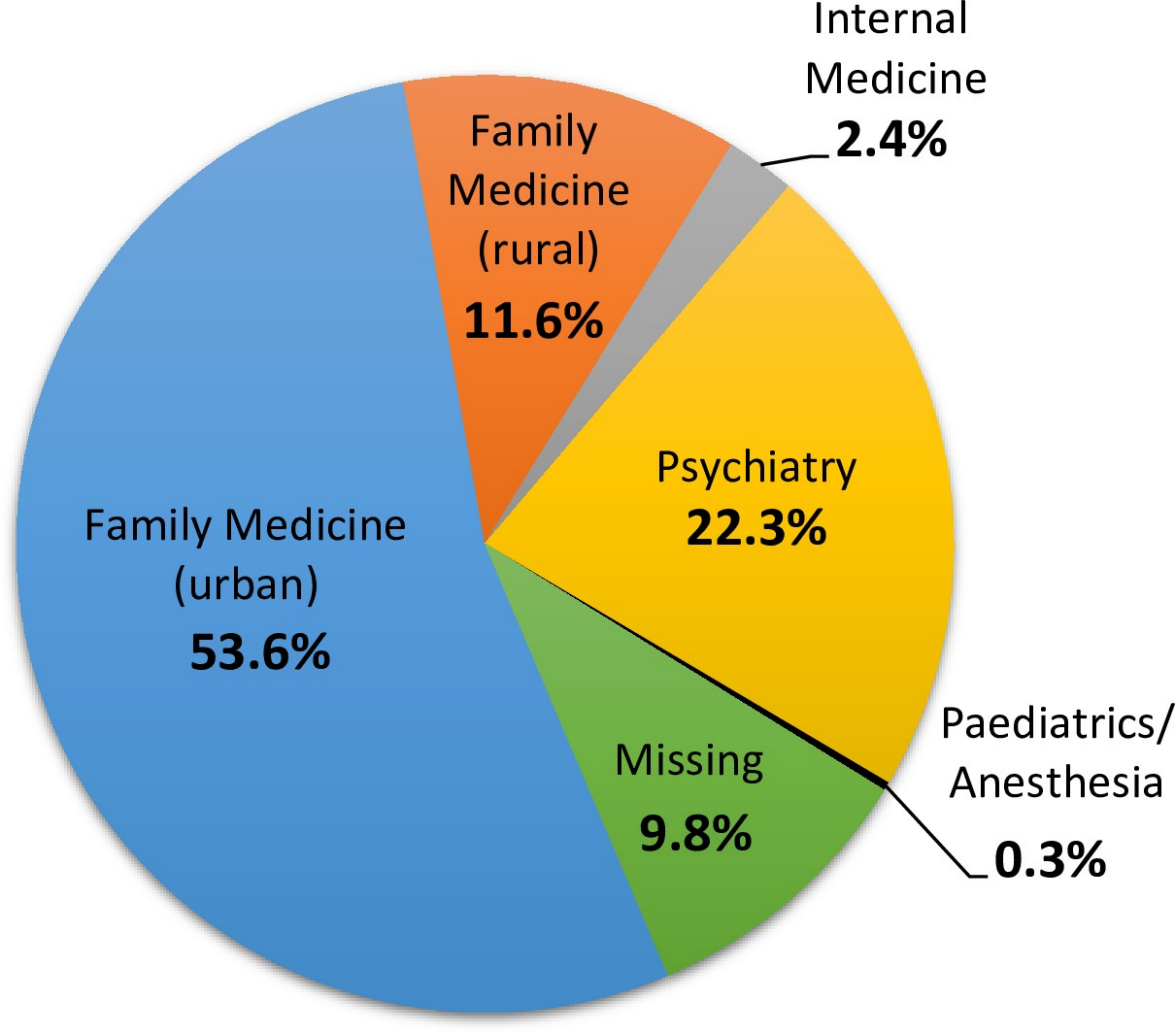
Physician specialties responsible for prescribing individuals’ first alcohol use disorder medication. Index dates 1996/97 to 2014/15.

**Fig 2:**
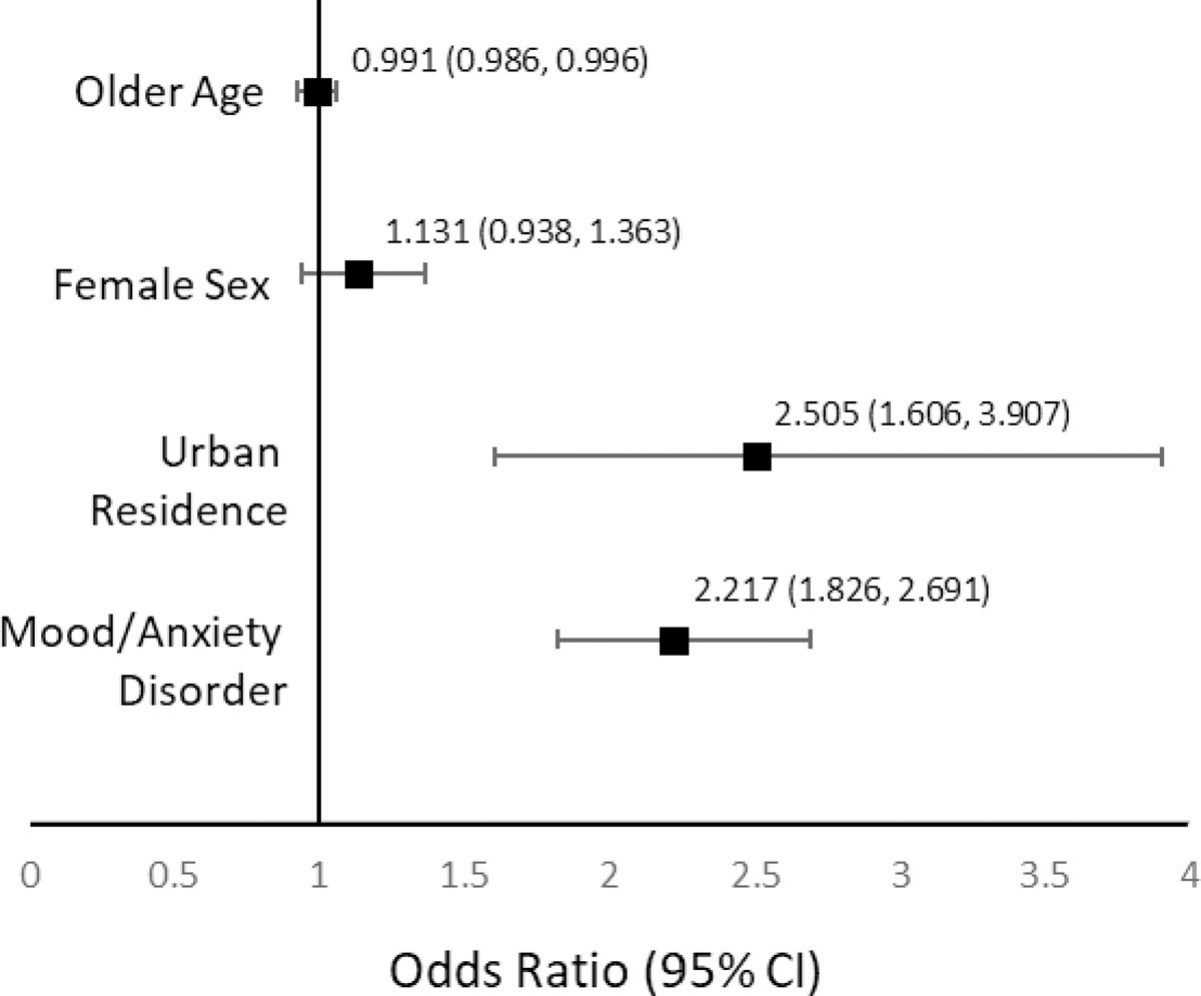
Variables predicting pharmacotherapy use among individuals with alcohol use disorder. Odds Ratio with 95% CI.

**Fig 1** shows that the majority of prescriptions for AUD medications came from family physicians practicing in urban settings, followed by psychiatrists and family physicians in rural settings. **Fig 2** presents the results of the hierarchical regression model, showing which variables were important predictors of individuals with AUD receiving pharmacotherapy. These findings indicate that individuals with AUD were more likely to fill an AUD prescription medication if they lived in an urban setting or had a comorbid mood or anxiety disorder diagnosis. Older individuals had reduced odds of receiving an AUD medication. Sex was not a significant predictor of receiving a prescription medication for AUD.

## Discussion

In the general population of Manitoba, only 1.3% of individuals diagnosed with AUD filled a prescription for an AUD medication over the course of the 20-year study period; naltrexone was the medication most commonly prescribed. This finding clearly highlights the profound underutilization of evidence-based medications for AUD, and is consistent with other research on low prescribing rates of AUD medications among US veterans [20, 21], low income Canadians [23], and Australians [32]. Low prescribing rates, uptake and utilization of AUD medications is a multi-faceted and complex matter influenced by patient, physician, and health system factors, including perceived low patient demand for the treatment, lack of provider knowledge about pharmacotherapy for alcohol dependence, and lack of confidence in treatment efficacy [33, 34].

In our study, the majority of individuals who filled a prescription spanned ages 30 to 50 years, a lengthy span which reflects the lag time typically observed between AUD diagnosis and onset of treatment [32]. While AUD often begins in young adulthood, the wide range of ages among individuals being treated for AUD points to a considerable gap between diagnosis and treatment, highlighting a need for improved AUD recognition in primary care settings and more aggressive outreach before individuals with AUD begin to present in emergent care. Older individuals in our study were less likely to receive pharmacotherapy for AUD than the 30–50 year old group. A similar finding has been found in other studies of health service use and treatment for substance use among older adults, despite positive treatment outcomes [35, 36]. These findings point to an opportunity to improve access to care and broader treatment options for older patients with AUD.

The vast majority of patients with AUD who received pharmacotherapy lived in urban settings, perhaps reflecting better access to prescription medications for AUD in urban vs rural areas in Manitoba. Individuals with AUD living in urban settings were also evenly distributed across income quintiles. This is an interesting finding as previous research has shown that lower income individuals are more likely to receive treatment for AUD [37]. Our finding can perhaps be explained by the availability of full coverage for eligible medications for all Manitoba residents after an income-based deductible is met. Finally, those who received AUD medications were also more likely to have overall higher health resource utilization and, in the year before diagnosis of AUD, have higher rates of hospitalizations. These findings likely reflect the impact of AUD on an individual’s physical health, contributing to increased resource use and hospitalization prior to pharmacotherapy being initiated, and are corroborated by a number of other studies [12, 30, 38].

In Manitoba, the majority of AUD medication prescriptions were provided by family physicians practicing in urban settings, with psychiatrists being the second most frequent prescribers. These results are in contrast to other studies of AUD medication prescribing, which found that psychiatrists are more likely than other medical specialties to use pharmacotherapy in the general population of individuals with AUD [34, 39]. Research from the US Veteran’s Health Administration has also shown that clinicians with dedicated training in addictions or physicians working at specialized addiction treatment centres tend to use pharmacotherapy at a higher rate than family physicians. However, in Manitoba, a large portion of addiction care is provided by family physicians, which may partially explain why they were the specialty most likely to prescribe AUD medications in our study. Overall, prescription rates for AUD medications were quite low, warranting improved education for physicians about the pharmacological options available to individuals with AUD. Family medicine, with its high prevalence of collaborative care models, is an ideal setting in which to initiate pharmacological treatment for these patients [40].

Our study found that individuals with AUD were more likely to be prescribed an AUD medication if they had a co-morbid mood or anxiety disorder diagnosis. This finding aligns with studies of co-morbidities among US veterans [20, 21]. Patients with a co-occurring disorder are more likely to present to specialty psychiatric care, and thus may benefit from psychiatrists’ increased knowledge of and/or experience with AUD medications. Furthermore, the efficacy of naltrexone in combination with the antidepressant sertraline has been demonstrated in a population with co-occurring AUD and depression [41]. Sex was not associated with receiving an AUD medication prescription, which is in keeping with research from Ontario, Canada [23].

### Strengths & limitations

Strengths of this study include the use of the Manitoba Population Research Data Repository, a comprehensive whole-population database of the residents of Manitoba, Canada. The use of administrative data overcomes limitations of recall bias and loss to follow-up. The DPIN database used in this study captures over 90% of prescriptions filled in community pharmacies in Manitoba and provides an accurate retrospective profile of individuals diagnosed with AUD and their medication prescriptions. Our study links data over 20 years, allowing for a robust analysis of predictive factors for receiving an AUD prescription medication.

Our study also has some recognized limitations. The administrative data only provide information on prescriptions filled at the pharmacy, and as such we do not have data on actual patient consumption. The number of prescriptions written by physicians may be underestimated in our study, since some prescriptions were likely generated but not filled by patients. As well, the DPIN database does not capture medications prescribed to patients in hospitals or penitentiaries–it is conceivable that some patients may have received an AUD medication while admitted that they discontinued once they were living in the community again. Off-label medications used for AUD, such as topiramate, were not included in the analysis; however, excluding them may have caused us to underestimate the proportion of individuals with AUD receiving pharmacotherapy. Information on non-drug interventions for managing AUD were not available in the administrative data.

## Conclusion

This large population-based longitudinal cohort study examined receipt of naltrexone, acamprosate, and disulfiram treatment among individuals with an AUD diagnosis in Manitoba, Canada. Despite established evidence for pharmacotherapeutic treatment of AUD, these medications were profoundly underutilized, potentially contributing to considerable public health harms for individuals, families and society at large. Future research in this area should focus on medication adherence and time to prescriptions following diagnosis in the general population, as well as specific barriers preventing pharmacotherapy treatment for individuals with AUD.

## Supporting information

S1 Table ICD codes for diagnosis of alcohol use disorder. (DOCX)

S2 Table ICD codes searched for comorbidities in individuals with alcohol use disorder. (DOCX)

S3 Table Diagnosis of comorbid mood or anxiety disorders in individuals with alcohol use disorder. (DOCX)

S4 Table ATC codes for prescriptions filled within one year of alcohol use disorder diagnosis. (DOCX)

## References

[1] Grant BF, Goldstein RB, Saha TD, Chou SP, Jung J, Zhang H, et al. Epidemiology of DSM-5 Alcohol Use Disorder: Results From the National Epidemiologic Survey on Alcohol and Related Conditions III. JAMA Psychiatry. 2015;72(8):757. doi: 10.1001/jamapsychiatry.2015.0584 26039070 PMC5240584

[2] World Health Organization. Global Status Report on Alcohol and Health 2018. Geneva; 2018.

[3] Chrystoja BR, Monteiro MG, Owe G, Gawryszewski VP, Rehm J, Shield K. Mortality in the Americas from 2013 to 2015 resulting from diseases, conditions and injuries which are 100% alcohol-attributable. Addiction. 2021; doi: 10.1111/add.15475 33844362

[4] Shield K, Manthey J, Rylett M, Probst C, Wettlaufer A, Parry CDH, et al. National, regional, and global burdens of disease from 2000 to 2016 attributable to alcohol use: a comparative risk assessment study. Lancet Public Heal. 2020 Jan 1;5(1):e51–61. 10.1016/S2468-2667(19)30231-2 31910980

[5] Rehm J, Shield KD, Weiderpass E. Alcohol consumption. A leading risk factor for cancer. Vol. 331, Chemico-Biological Interactions. Elsevier Ireland Ltd; 2020. 10.1016/j.cbi.2020.109280 33010221

[6] Connor J. Alcohol consumption as a cause of cancer. Addiction. 2017;112(2):222–8. doi: 10.1111/add.13477 27442501

[7] Hafekost K, Lawrence D, O’Leary C, Bower C, Semmens J, Zubrick R. Maternal Alcohol Use Disorder and Risk of Child Contact with the Justice System in Western Australia: A Population Cohort Record Linkage Study. Alcohol Clin Exp Res. 2017;41(8):1452–60. doi: 10.1111/acer.13426 28641361 PMC5575459

[8] Elliott JC, Stohl M, Wall MM, Keyes KM, Skodol AE, Eaton NR, et al. Childhood maltreatment, personality disorders and 3-year persistence of adult alcohol and nicotine dependence in a national sample. Addiction. 2016 May 1;111(5):913–23. doi: 10.1111/add.13292 26714255 PMC4826838

[9] Elliott JC, Stohl M, Wall MM, Keyes KM, Goodwin RD, Skodol AE, et al. The risk for persistent adult alcohol and nicotine dependence: The role of childhood maltreatment. Addiction. 2014;109(5):842–50. doi: 10.1111/add.12477 24401044 PMC3984602

[10] Dostanic N, Djikanovic B, Jovanovic M, Stamenkovic Z, Đeric A. The Association Between Family Violence, Depression and Anxiety Among Women Whose Partners Have Been Treated for Alcohol Dependence. J Fam Violence. 2021; doi: 10.1007/s10896-020-00238-1 33424110 PMC7778496

[11] Wilson SR, Lubman DI, Rodda S, Manning V, Yap MBH. The personal impacts of having a partner with problematic alcohol or other drug use: descriptions from online counselling sessions. Addict Res Theory. 2018 Jul 4;26(4):315–22.

[12] Myran DT, Hsu AT, Smith G, Tanuseputro P. Rates of emergency department visits attributable to alcohol use in Ontario from 2003 to 2016: a retrospective population-level study. CMAJ. 2019;191(29):E804–10. doi: 10.1503/cmaj.181575 31332048 PMC6645924

[13] Guitart AM, Espelt A, Castellano Y, Suelves JM, Villalbí JR, Brugal MT. Injury-Related Mortality Over 12 Years in a Cohort of Patients with Alcohol Use Disorders: Higher Mortality Among Young People and Women. Alcohol Clin Exp Res. 2015;39(7):1158–65. doi: 10.1111/acer.12755 26033536

[14] Hulme J, Sheikh H, Xie E, Gatov E, Nagamuthu C, Kurdyak P. Mortality among patients with frequent emergency department use for alcohol-related reasons in Ontario: A population-based cohort study. CMAJ. 2020 Nov 23;192(47):E1522–31. doi: 10.1503/cmaj.191730 33229348 PMC7721258

[15] Martinez CP, Vakkalanka P, Ait-Daoud N. Pharmacotherapy for alcohol use disorders: Physicians’ perceptions and practices. Front Psychiatry [Internet]. 2016 Nov 14 [cited 2021 Jun 7];7(NOV). Available from: /pmc/articles/PMC5108053/10.3389/fpsyt.2016.00182 PMC5108053 27895598

[16] Jonas DE, Amick HR, Feltner C, Bobashev G, Thomas K, Wines R, et al. Pharmacotherapy for Adults With Alcohol Use Disorders in Outpatient Settings. JAMA. 2014;311(18):1889. doi: 10.1001/jama.2014.3628 24825644

[17] Goh ET, Morgan MY. Review article: pharmacotherapy for alcohol dependence–the why, the what and the wherefore. Aliment Pharmacol Ther. 2017;45(7):865–82. doi: 10.1111/apt.13965 28220511

[18] Kranzler H, Soyka M. Diagnosis and pharmacotherapy of alcohol use disorder: A review. JAMA. 2018;320:815–24. doi: 10.1001/jama.2018.11406 30167705 PMC7391072

[19] Castrén S, Mäkelä N, Alho H. Selecting an appropriate alcohol pharmacotherapy: Review of recent findings. Vol. 32, Current Opinion in Psychiatry. Lippincott Williams and Wilkins; 2019. p. 266–74. doi: 10.1097/YCO.0000000000000512 30973369

[20] Iheanacho T, Issa M, Marienfeld C, Rosenheck R. Use of naltrexone for alcohol use disorders in the Veterans’ Health Administration: A national study. Drug Alcohol Depend. 2013;132(1–2):122–6. doi: 10.1016/j.drugalcdep.2013.01.016 23434041

[21] Harris A, Oliva E, Bowe T, Humphreys K, Kivlahan D, Trafton J. Pharmacotherapy of Alcohol Use Disorders by the Veterans Health Administration: Patterns of Receipt and Persistence. Psychiatr Serv. 2012;63(7):679–85. doi: 10.1176/appi.ps.201000553 22549276

[22] Finlay AK, Ellerbe LS, Wong JJ, Timko C, Rubinsky AD, Gupta S, et al. Barriers to and facilitators of pharmacotherapy for alcohol use disorder in VA residential treatment programs. J Subst Abuse Treat [Internet]. 2017 Jun 1 [cited 2021 Jun 7];77:38–43. Available from: /pmc/articles/PMC5467688/ doi: 10.1016/j.jsat.2017.03.005 28476269 PMC5467688

[23] Spithoff S, Gomes T, Martins D, Singh S, Spithoff S, Martins D. First-line medications for alcohol use disorders among public drug plan beneficiaries in Ontario. Can Fam Physician. 2017;63(5):277–83. 28500210 PMC5429069

[24] Oreopoulos P, Stabile M, Walld R, Roos L. Short-medium, and long term consequences of poor infant health: An analysis using siblings and twins. J Hum Resour. 2008;43:88–138.

[25] O’Grady K, Deussing M, Scerbina T, Fund K, Muhe N. Measuring up: Canadian Results of the OECD PISA study. http://cmec.ca/Publications/Lists/Publications/Attachments/365/Book_PISA2015_EN_Dec5.pdf. Council of Ministers of Education, Canada; 2016.

[26] Roos LL, Gupta S, Soodeen R, Jebamani L. Data quality in an information-rich environment: Canada as an example. Can J Aging. 2005;24(Suppl 1):153–70. 16080132 10.1353/cja.2005.0055

[27] Roos LL, Menec V, Currie RJ. Policy analysis in an information-rich environment. Soc Sci Med. 2004 Jun;58(11):2231–41. doi: 10.1016/j.socscimed.2003.08.008 15047080

[28] The Johns Hopkins University Bloomberg School of Public Health. The Johns Hopkins ACG Case-Mix System Documentation & Application Manual, Version 10. Weiner JP, editor. Baltimore, MD: Johns Hopkins University; 2001.

[29] Shield K, Parry C, Rehm J. Chronic diseases and conditions related to alcohol use. Alcohol Res Curr Rev. 2014;35(2):155–71. 10.35946/arcr.v35.2.06 PMC3908707 24881324

[30] Miquel L, Gual A, Vela E, Lligoña A, Bustins M, Colom J, et al. Alcohol consumption and inpatient health service utilization in a cohort of patients with alcohol dependence after 20 years of follow-up. Alcohol Alcohol. 2017;52(2):227–33. doi: 10.1093/alcalc/agw075 28182212

[31] Rehm J, Shield K. Global alcohol-attributable deaths from cancer, liver cirrhosis, and injury in 2010. Alcohol Res Curr Rev. 2013;35(2):174–83. 10.35946/arcr.v35.2.07 PMC3908708 24881325

[32] Morley KC, Logge W, Pearson SA, Baillie A, Haber PS. National trends in alcohol pharmacotherapy: Findings from an Australian claims database. Drug Alcohol Depend. 2016;166:254–7. doi: 10.1016/j.drugalcdep.2016.06.027 27394934

[33] Harris A, Ellerbe L, Reeder RN, Bowe T, Gordon AJ, Hagedorn H, et al. Pharmacotherapy for alcohol dependence: perceived treatment barriers and action strategies among Veterans Health Administration service providers. Psychol Serv. 2013;10(4):410–9. doi: 10.1037/a0030949 23356858

[34] Ponce Martinez C, Vakkalanka P, Ait-daoud N. Pharmacotherapy for Alcohol Use Disorders: Physicians ‘ Perceptions and Practices. Front Psychiatry. 2016;7(November):1–6. 27895598 10.3389/fpsyt.2016.00182 PMC5108053

[35] Dauber H, Pogarell O, Kraus L, Braun B. Older adults in treatment for alcohol use disorders: Service utilisation, patient characteristics and treatment outcomes. Subst Abus Treat Prev Policy [Internet]. 2018 Nov 6 [cited 2021 Jun 15];13(1):1–10. Available from: doi: 10.1186/s13011-018-0176-z 30400930 PMC6220462

[36] Kuerbis A, Sacco P, Blazer DG, Moore AA. Substance Abuse Among Older Adults [Internet]. Vol. 30, Clinics in Geriatric Medicine. W.B. Saunders; 2014 [cited 2021 Jun 15]. p. 629–54. Available from: /pmc/articles/PMC4146436/ doi: 10.1016/j.cger.2014.04.008 25037298 PMC4146436

[37] Ilgen MA, Price AM, Burnett-Zeigler I, Perron B, Islam K, Bohnert ASB, et al. Longitudinal predictors of addictions treatment utilization in treatment-na??ve adults with alcohol use disorders. Drug Alcohol Depend. 2011;113(2–3):215–21. doi: 10.1016/j.drugalcdep.2010.08.006 20828944 PMC3005968

[38] Cherpitel CJ. Changes in substance use associated with emergency room and primary care services utilization in the United States general population: 1995–2000. Am J Drug Alcohol Abuse. 2003;29(4):789–802. doi: 10.1081/ada-120026261 14713140

[39] Mark Tami L.; Kassed Cheryl A.; Vandivort-Warren Rita; Levit Katharine R.; Kranzler HR. Alcohol and Opioid Dependence Medications: Prescription Trends, Overall and by Physician Specialty. Drug Alcohol Depend. 2009;99(1–3):345–9. doi: 10.1016/j.drugalcdep.2008.07.018 18819759 PMC3166770

[40] Watkins KE, Ober AJ, Lamp K, Lind M, Setodji C, Osilla KC, et al. Collaborative Care for Opioid and Alcohol Use Disorders in Primary Care. JAMA Intern Med. 2017;177(10):1480–8. doi: 10.1001/jamainternmed.2017.3947 28846769 PMC5710213

[41] Pettinati HM, Oslin DW, Kampman KM, Dundon WD, Xie H, Gallis TL, et al. A double blind, placebo-controlled trial that combines sertraline and naltrexone for treating co-occurring depression and alcohol dependence. Am J Psychiatry. 2010;167(6):668. doi: 10.1176/appi.ajp.2009.08060852 20231324 PMC3121313

