# Supplementary material for: Use of pharmacotherapy for alcohol use disorder in Manitoba, Canada: A whole-population cohort study
Source: PLoS One. 2021 Sep 3;16(9):e0257025. doi: 10.1371/journal.pone.0257025 (PMC8415582; doi:10.1371/journal.pone.0257025)
Supplement: S1 Table — (DOCX) [file pone.0257025.s001.docx]

| **S1** **Table. ICD Codes for Diagnosis of Alcohol Use Disorder** | |
| --- | --- |
| **ICD-9-CM Code** | **Diagnosis** |
| ICD-9-CM 291 | Alcohol Abuse |
| 303 | Alcohol Dependence Syndrome |
| 305 | Alcohol Abuse |
| 357.5 | Alcoholic Polyneuropathy |
| 425.5 | Alcoholic Cardiomyopathy |
| 535.3 | Alcoholic Gastritis |
| 571.0-571.4 | Alcoholic Fatty Liver |
|  | Acute Alcoholic Hepatitis |
|  | Alcoholic Cirrhosis |
|  | Alcoholic Liver Damage Unspecified |
|  | Chronic Hepatitis - excludes viral |
| **ICD-10-CA Code** | **Diagnosis** |
| F10 | Mental and Behavioral Disorders due to use of Alcohol |
| G31.2 | Degeneration of Nervous System due to Alcohol |
| G62.1 | Alcoholic Polyneuropathy |
| I42.6 | Alcoholic Cardiomyopathy |
| K29.2 | Alcoholic Gastritis |
| K70 | Alcoholic Liver Disease |
| K85.2, K86.0 | Alcoholic-Induced Pancreatitis |
