# Supplementary material for: Use of pharmacotherapy for alcohol use disorder in Manitoba, Canada: A whole-population cohort study
Source: PLoS One. 2021 Sep 3;16(9):e0257025. doi: 10.1371/journal.pone.0257025 (PMC8415582; doi:10.1371/journal.pone.0257025)
Supplement: S2 Table — (DOCX) [file pone.0257025.s002.docx]

| **S2 Table.** **ICD Codes Searched for Comorbidities in Individuals with Alcohol Use Disorder** | |
| --- | --- |
| **Personality Disorders (1+ Dx)** | |
| **ICD-9-CM** |  |
| 301 | Personality disorders |
| **ICD-10-CA** |  |
| F21 | Schizotypal disorder |
| F60 | Specific personality disorders |
| F61 | Mixed and other personality disorders |
| F62 | Enduring personality changes, not attributable to brain damage and disease |
| F69 | Unspecified disorder of adult personality and behaviour |
| **Psychoses (1+ Dx)** | |
| **ICD-9-CM** |  |
| 295 | Schizophrenic disorders |
| 297 | Delusional disorders |
| 298 | Other nonorganic psychoses |
| **ICD-10-CA** |  |
| F11.5 | Mental and behavioural disorders due to use of opioids, psychotic disorder |
| F12.5 | Mental and behavioural disorders due to use of cannabinoids, psychotic disorder |
| F13.5 | Mental and behavioural disorders due to use of sedatives or hypnotics, psychotic disorder |
| F14.5 | Mental and behavioural disorders due to use of cocaine, psychotic disorder |
| F15.5 | Mental and behavioural disorders due to use of other stimulants including caffeine, psychotic disorder |
| F16.5 | Mental and behavioural disorders due to use of hallucinogens, psychotic disorder |
| F17.5 | Mental and behavioural disorders due to use of tobacco, psychotic disorder |
| F18.5 | Mental and behavioural disorders due to use of volatile solvents, psychotic disorder |
| F19.5 | Mental and behavioural disorders due to multiple drug use and use of psychoactive substances, psychotic disorder |
| F20 | Schizophrenia |
| F21 | Schizotypal disorder |
| F22 | Persistent delusional disorders |
| F23 | Acute and transient psychotic disorders |
| F24 | Induced delusional disorder |
| F25 | Schizoaffective disorders |
| F28 | Other nonorganic psychotic disorders |
| F29 | Unspecified nonorganic psychosis |
| **Drug Abuse (1+ Dx)** | |
| **ICD-9-CM** | |
| 292 | Drug-induced mental disorders |
| 304 | Drug dependence |
| 305.1-305.9 | Nondependent abuse of drugs |
| **ICD-10-CA** | |
| F11 | Mental and behavioural disorders due to use of opioids |
| F12 | Mental and behavioural disorders due to use of cannabinoids |
| F13 | Mental and behavioural disorders due to use of sedatives or hypnotics |
| F14 | Mental and behavioural disorders due to use of cocaine |
| F15 | Mental and behavioural disorders due to use of other stimulants, including caffeine |
| F16 | Mental and behavioural disorders due to use of hallucinogens |
| F17 | Mental and behavioural disorders due to use of tobacco |
| F18 | Mental and behavioural disorders due to use of volatile solvents |
| F19 | Mental and behavioural disorders due to multiple drug use and use of other psychoactive substances |
| F55 | Abuse of non-dependence-producing substances |
