# Supplementary material for: Use of pharmacotherapy for alcohol use disorder in Manitoba, Canada: A whole-population cohort study
Source: PLoS One. 2021 Sep 3;16(9):e0257025. doi: 10.1371/journal.pone.0257025 (PMC8415582; doi:10.1371/journal.pone.0257025)
Supplement: S3 Table — (DOCX) [file pone.0257025.s003.docx]

| **S3 Table.** **Diagnosis of Comorbid Mood or Anxiety Disorders in Individuals with Alcohol Use Disorder** | |
| --- | --- |
| **Algorithm for Diagnosis** | **Diagnostic Codes** |
| Algorithm 1 | 1+ hospitalization with diagnosis codes:  ICD-9-CM 296.1-296.8, 300.0, 300.2, 300.3, 300.4, 300.7, 309 or 311; ICD-10-CA F31-F33, F341, F38.0, F38.1, F40, F41.0, F41.1, F41.2, F41.3, F41.8, F41.9, F42, F43.1, F43.2, F43.8, F53.0, or F93.0 |
| Algorithm 2 | 1+ hospitalization with diagnosis codes:  ICD-9-CM 300; ICD-10-CA F32, F341, F40, F41, F42, F44, F45.0, F45.1, F45.2, F48, F68.0 or F99  AND  One or more prescriptions for an antidepressant or mood stabilizer: ATC codes N05AN01, N05BA, N06A, N05BE01 |
| Algorithm 3 | 1+ physician visit with diagnosis codes:  ICD-9-CM 296, 311 |
| Algorithm 4 | 1+ physician visits with a diagnosis code:  ICD-9-CM 300  AND  One or more prescriptions for an antidepressant or mood stabilizer: ATC codes N05AN01, N05BA, N06A, N05BE01 |
| Algorithm 5 | 3+ physician visits with a diagnosis code:  ICD-9-CM 300, 309 |
| Administrative data used to measure these diagnoses were from the five years before index date. | |
