# Supplementary material for: Use of pharmacotherapy for alcohol use disorder in Manitoba, Canada: A whole-population cohort study
Source: PLoS One. 2021 Sep 3;16(9):e0257025. doi: 10.1371/journal.pone.0257025 (PMC8415582; doi:10.1371/journal.pone.0257025)
Supplement: S4 Table — (DOCX) [file pone.0257025.s004.docx]

| **S4 Table.** **ATC Codes for Prescriptions Filled Within One Year of Alcohol Use Disorder Diagnosis** | |
| --- | --- |
| **ATC Code** | **Prescription Medication** |
| N07BB01 | Disulfiram |
| N07BB03 | Acamprosate |
| N07BB04 | Naltrexone |
| N06AB | SSRI Antidepressants |
| N06AA | TCA Antidepressants |
| N06AF, N06AG, N06AX | Other Antidepressants |
| N05BA, N05C, N03AE01 | Sedatives/Anxiolytics |
| N05BE01 | Buspirone |
| N05A (excl. N05AN) | Antipsychotics |
| N03AX12, N03AX16 | Gabapentin/Pregabalin |
| N03AX11 | Topiramate |
| N03AG01, N03AG02 | Valproic Acid |
| N03AF01 | Carbamazepine |
| N03AX09 | Lamotrigine |
| N05AN | Lithium |
| M03B | Skeletal Muscle Relaxants |
| N02A | Opioids |
